# Comparing short term and sustained effects of two strategies to improve nurses’ adherence with hand hygiene prescriptions: a cluster randomised trial

**DOI:** 10.1186/1753-6561-5-S6-P119

**Published:** 2011-06-29

**Authors:** A Huis, L Schoonhoven, R Grol, R Donders, M Hulscher, T van Achterberg

**Affiliations:** 1 IQ Healthcare, Radbound University Nijmegen Medical Centre, Nijmegen, Netherlands

## Introduction / objectives

Improving hand hygiene (HH) compliance is still a major challenge. We compared a literature based 'state of the art' strategy with a 'team directed' strategy, based on social influence and leadership, on their effectiveness on HH compliance.

## Methods

We undertook a cluster randomised trial with inpatient wards as the unit of randomisation. The 'state of the art' strategy (SAS) included education, reminders, feedback and targeting adequate products and facilities. The 'team directed' strategy (TD) also contained activities based on social influence and leadership, gaining active commitment and initiative of ward management, modelling by informal leaders, and setting norms and targets within the team. Strategies were delivered during a period of 6 months. Measurements took place directly before and after strategy delivery and 6 months later. The effects were evaluated on an intention-to-treat basis by comparing the post-strategyÂ hand hygieneÂ compliance rates with the baseline rates. Multilevel analysis was applied to compensate for the clustered nature of the data by using mixed linear modelling techniques.

## Results

The SAS showed a short-term improvement of 19,6% and a long term improvement of 23,7%. The improvement for the TD was 33,7% (short term) and 33.1% (long term). The difference between TD and SAS showed an Odds Ratio of 1.641 (p<0.001) in favour of the 'team directed' strategy.

## Conclusion

Both the 'state of the art' strategy and the 'team directed' strategy successfully improved hand hygiene compliance, but the ‘team directed' strategy showed even better results. The methodology of this ‘team directed’ strategy can also be used to improve team performance on other patient safety issues.

## Disclosure of interest

None declared.

